# Get in the Virtual Hole! Examination of Gaze and Performance of Experts, Athletes, and Novices While Putting in Virtual Reality and in the Real‐World

**DOI:** 10.1002/ejsc.70049

**Published:** 2025-08-30

**Authors:** Jayke B. Bennett, David L. Neumann, Matthew J. Stainer

**Affiliations:** ^1^ School of Applied Psychology Griffith University Brisbane Queensland Australia

**Keywords:** golf putting, psychological fidelity, quiet eye, virtual reality, VR golf putting

## Abstract

Virtual reality (VR) offers opportunities to train and assess visuomotor skills and sports performance in controlled, reproducible contexts, supporting innovation in research and training. However, VR imposes unique sensory demands that may disrupt movement coordination, task performance, and potential skill learning. This study used putting performance assessment and eye‐tracking to examine visuomotor coordination and performance in VR versus the real world, and whether effects varied by task expertise and athlete skill. Novice undergraduates (*n* = 44), national/international athletes in sports other than golf (*n* = 14), and expert golfers (*n* = 5) completed 30 putts in both settings while eye‐gaze and putting outcomes were measured. Novices were divided into low performing (LPN) and high performing (HPN) based on real‐world performance, while athletes and experts were not. More putts were holed in the real‐world than VR across expertise groups, while experts holed many more putts in the real‐world than athletes, HPN, and LPN, while athletes and HPN holed more putts than LPN. There were no differences between groups in VR holed putts. Putting radial error (RE) was much lower for experts, and moderately lower for athletes and HPN than LPN in the real‐world. Experts had moderately lower RE than HPN in VR. Quiet Eye (QE), the final fixation prior to movement execution, predicted reduced RE for experts in the real‐world but not in VR. Visuomotor co‐ordination in VR may be disrupted. VR training environments may need adjustment to address visuomotor differences and should be designed to deliver feedback consistent with real‐world performance expectations.

## Highlights

- Superior performance is found for experts when putting in the real‐world compared to athletes, high performing novices (HPN) and low performing novices (LPN), whereas a performance advantage in VR is found for experts only compared to HPN. Putting performance was improved for all groups in the real‐world than in VR.
- Only experts showed similar performance across any putting metric between the real‐world and VR, namely putting line. This may suggest that an advantage of expertise may be consistency in some aspects of performance across contexts, potentially indicating that some skills may generalize while others may not.
- Holding a longer QE is found to benefit performance for experts only, an effect found in the real‐world but not in VR. Coupled with difference in eye‐gaze behaviours between VR and the real‐world, these findings may suggest that visuomotor coordination in VR may be disrupted compared to the real‐world.
- If using VR to train visuomotor skills, a recommendation may be to use VR to train skills that may be difficult to effectively isolate and train as compared to the real‐world, instead of using VR as an alternative visuomotor practice environment.

## 1 Introduction

Virtual reality (VR) is a computerised environment which provides sensory information to a user to create immersion and a subjective sense of presence and allows users to interact with the environment (Neumann et al. 2018). In sport, VR can help train motor skills, expose athletes to performance under pressure, or allow for pattern‐recognition expertise to develop through repetition of in‐game scenarios with real‐time feedback (Akbaş et al. 2019; Bird 2020). VR may also be useful for performance evaluation, as coaches, players or researchers can extract, analyse, and apply performance‐related data insights in real‐time feedback, which can assist athletic development (Bird 2020). Using VR for athletic training somewhat assumes that visuomotor co‐ordination in VR is like the real‐world; however, the extent to which VR performance is due to motor skill execution, the demands of VR itself (e.g., perceptual) or a combination of both, requires further clarification (Michalski et al. 2019). The purpose of the present study was to compare visuomotor co‐ordination between VR and the real‐world across people with varying levels of task and motor expertise. The aim was to clarify how VR may impact upon visuomotor performance using a golf putting task, examine what these differences to the real‐world may be, and whether the suitable use of VR for training may be based on task expertise, general motor skills, or a combination of these factors.

### 1.1 Performance of Visuomotor Skills in VR

VR, like the real‐world, preserves the *perception‐action* loop, meaning that athletes can receive real‐time feedback about their movement and its effects (D. J. Harris et al. 2019). One such example is VR golf, where users can putt the ball towards the hole while being able to see the clubhead movement, and receive visual feedback on putt outcome (D. J. Harris, Buckingham, et al. 2020). However, when using Head Mounted Display (HMD) VR, there are differences to the real world in how visual information is presented to the eyes (Düking et al. 2018). The differences have been shown in experimental settings to lead to the misperception of object size and spatial characteristics (e.g., depth perception), which may contribute to changes in how subsequent visuomotor actions are performed (Whitwell and Buckingham 2013). In addition, there can be a lack of haptic end‐point feedback when completing a task in VR, resulting in lower velocity movements and gestures compared to the real‐world, culminating in longer grasping times and movement sequences (Wijeyaratnam et al. 2019). Individually and in combination, these differences may lead to changes in sensory processing that is thought to contribute to perceptual uncertainty, and an unconscious effort to programme movement in a different way to meet novel task demands—which is typically inconsistent to the real‐world (Giesel et al. 2020). Because of these factors, VR may be a more difficult environment for people to perform in, and to adapt and refine their motor actions compared to the real‐world, which has flow on effects for quality of movement (Wang et al. 2023), and potentially for how skills are learnt in VR.

Although literature shows differences to visual demands and visually guided behaviour in VR as compared to the real‐world, there is evidence that some tasks learnt in VR may confer real‐world performance benefits (D. J. Harris, Buckingham, et al. 2020; Michalski et al. 2019; Tirp et al. 2015), and that experts in the real‐world may still show an expert performance advantage in VR relative to novices (D. J. Harris et al. 2021; Wood et al. 2020). However, it is critical to also note that some experiments have shown that VR performance, irrespective of whether someone is a task novice or an expert, may still be poorer than in the real‐world (D. J. Harris et al. 2021). This may suggest a potential issue in the generalizability of using VR for sports training, defined as the extent of which motor, cognitive and mental skills, strategies, and tactics can be used in untrained or unfamiliar situations (Richlan et al. 2023). Key tenets of generalizability is for who a specific training protocol is appropriate for (e.g., novices, athletes, and experts), what the tasks to be trained are (e.g., tactics and strategies or visuomotor development), if these interventions are efficacious in improving the targeted skill or behaviour, and whether the task is equivalent across performance contexts (such as across VR and the real‐world). Prior to the implementation of a specific training protocol in VR, it is important to consider that VR training may not always translate to real‐world improvements within a sporting context (D. J. Harris, Buckingham, et al. 2020).

Are the expert performance advantages over less skilled individuals in VR due to specialised expertise in that particular sport or visuomotor task (e.g., expertise in golf putting)? Or could this effect also be explained through a general ability for people of high athletic skill to maintain a performance advantage under novel task demands (Ericsson and Lehmann 1996; Williams and Ford 2008; Wood et al. 2020)? It may be plausible that athletic skill only takes you so far when adapting to a VR environment, and expert task knowledge is required to mitigate some of the detrimental effects of VR on skill performance. Considering cognitive load theory, for example, where VR demands may increase cognitive load (Juliano et al. 2022), experts may have an increased capacity to adapt to VR environments because their performance of the motor task expends less cognitive resources compared to novices. Even when in VR, expert performance is thus still within working memory capacity (Orru and Longo 2019). In this case, VR practice may theoretically be suitable for experts but not for novices on the singular dimension of cognitive load. Other aspects, such as behavioural and cognitive equivalence related to how people plan the task and perform a motor action, would still require verification to justify the appropriate use of VR for motor skill practice or for the training of visuomotor skills in VR environments for each sporting task.

Task experts may also have an advantage in regulating their attention on the specific motor task compared to people of intermediate skill levels (Amico and Schaefer 2022). A distinction then becomes whether people who are expert level performers in another sport are also able to regulate their attention more effectively than novices (which would speak to a cognitive dimension of fidelity), and whether performance may still be impaired through the combination of perceptual and motor learning demands when performing under novel constraints. Although VR is being increasingly used for motor skill learning and training (Akbaş et al. 2019; Bird 2020; Neumann et al. 2018), there remains a gap in understanding who can perform well within VR environments, and how task expertise, movement skill, and adaptation to VR, impact upon the suitability of using VR for skill practice. It is important to ensure that athletes are learning and refining their skills within contexts where their actions will provide similar task‐related feedback to the real‐world, particularly if a VR environment is being considered for training visuomotor skills to then be performed in the real‐world (Le Noury et al. 2021).

### 1.2 Eye‐Gaze: Evaluating Visuomotor Coordination in VR

One cluster of measures which intersects performance and expertise is gaze behaviour. Expert‐level athletes represent the pinnacle of hand–eye co‐ordination for their given sport, as their gaze behaviour is tuned to look in the right place (fixation location) at the right time (temporal sequence) for optimal task success (J. Vickers 2007). Compared to novices, elite athletes can more effectively interpret task‐specific perceptual cues, and tend to make fewer fixations, but of longer duration, and superior spatial accuracy (Mann et al. 2007). Expert athletes also look earlier than novices towards areas of interest (AOI; including looking at blank space where a ball *will* arrive in the future; Land and McLeod 2000; Hayhoe 2017), which helps the optimal movement to be programmed and executed under changing task constraints (Hayhoe 2017; Land and McLeod 2000; Rienhoff et al. 2016). One hallmark of visuomotor expertise is Quiet Eye (QE), the final fixation on an AOI prior to the critical movement in a task, which lasts for at least 100 ms and can extend beyond movement completion (J. N. Vickers and Kopp 2016). Experts hold a longer QE than near‐elite counterparts and novices, but holding a longer QE is associated with improved performance irrespective of expertise (Lebeau et al. 2016). Selecting the appropriate task‐movement (Klostermann and Hossner 2018), use of automatic movement control processes, and quiescence (slowing of heart and respiration rate to allow the movement to smoothly occur against the body's natural movement from these processes), are thought to underpin the performance‐enhancing effects of QE (Moore et al. 2012), individually or in combination (Gonzalez et al. 2017).

QE and associated eye‐gaze behaviours have been extensively studied within the context of golf‐putting, where there is a well‐established pattern of visuomotor co‐ordination required for task success (Campbell and Moran 2014). Although gaze‐behaviour in general, including fixation location, duration, and sequence, provide insights into performance and expertise (Ziv and Lidor 2019), QE is a robust predictor of motor and task performance. Moreover, it is of particular interest due to its use within visuomotor training protocols such as Quiet Eye Training, which aims to teach novices or near‐elite athlete counterparts the QE fixation behaviour of an expert to improve motor skill performance (Lebeau et al. 2016). Typically, experts make fewer than three fixations towards the hole prior to setting when putting (Moore et al. 2012), and deploy a QE fixation which lasts around 2.5 s prior to backswing (J. N. Vickers 1992). Although there can be inter‐expert differences in visuomotor timing (Ziv and Lidor 2019), the tight hand–eye co‐ordination required for golf putting provides a framework to understand how visuomotor co‐ordination may be affected by the novel task demands of VR, and whether this affect is consistent across levels of task and motor expertise, providing insights to how cognition and performance may interact within VR and real‐world environments. A strength of evaluating putting performance is that it can also provide insights into angular accuracy (i.e., line of putt) and power used (i.e., length of putt) relative to overall error (i.e., radial error, distance between the ball and hole, RE), which offers nuance into how visuomotor variability may affect performance outcomes.

### 1.3 Present Study

The aim of the study was to compare visuomotor co‐ordination and putting performance in VR and the real‐world, to evaluate how differences may contribute to performance, and be related to expertise factors (such as task skill, general skill and ability to adapt). The following hypotheses were tested.

1. Expert golfers will have superior putting performance compared to athletes and novices in VR and in the real‐world environment, due to literature which suggests that expertise differences in motor performance can be preserved in VR (D. J. Harris et al. 2021; Wood et al. 2020).
2. Putting performance will be more accurate in the real‐world environment compared to VR, due to disruptions to visuomotor co‐ordination in VR (D. J. Harris et al. 2019; Valori et al. 2021).
3. Experts will demonstrate a longer QE than athletes and novices in VR and in the real‐world environment (Mann et al. 2007).

## 2 Method

### 2.1 Participants

Table 1 provides demographic details for the novice, athlete, and expert participants. The novice participants were further split into low performing novice (LPN) and high performing novice (HPN) groups. People were excluded if they had visual abnormalities (such as diplopia and strabismus) as determined by self‐report. Based on the combined large effect size difference on RE performance between VR and the real‐world for experts and novices (D. J. Harris et al. 2021), sufficient statistical power for Linear Mixed Model analyses with 30 putts in VR and in the real‐world was achieved at a sample of 5 participants per group (*α* = 0.05, *β* = 0.80), or at 20 total participants as calculated using the *simr* package (Green and MacLeod 2016).

**TABLE 1:** Demographic characteristics of the novice (including low performing and high performing subgroups), athlete, and expert participant samples.

Novice			Athlete	Expert
Demographic variable	Low performing	High performing		
*n*	22	22	14	5
Age (SD)	25.17 (8.37)	27.09 (8.52)	22.36 (4.83)	35.80 (19.82)
Male:female	8:14	13:9	7:7	5:0
Normal vision (%)	91.30	100.00	92.90	60.00
Corrected to normal vision (%)	8.70	0.00	7.10	40.00
VR use at all (%)	39.10	26.10	28.60	0.00
VR use for training (%)	0.00	0.00	0.00	0.00
VR use for gaming (%)	17.40	30.40	21.40	0.00
Putted right‐handed (%)	100.00	100.00	100.00	100.00

*Note:* Low Performing Novices (LPN) and High Performing Novices (HPN) hereafter.

*Novices*. Novice undergraduate students (*n* = 44) participated in exchange for course credit. The average age was 26.13 years (SD = 8.41). No novices were a national or international level athlete in a sport. Some had received golf‐training (8.7%), and had a stroke handicap (4.4%); however, none played competitively (0%). Both novices with a stroke handicap exceeded 25, where 20 can be considered at a novice skill level (Merry et al. 2022), meaning that all participants met the definition of a novice golfer (Moore et al. 2012).

*Athletes*. National and/or international athletes in a sport other than golf (*n* = 14) participated in exchange for course credit (71.4%) or $50 AUD worth of gift‐cards (28.6%). Some athletes previously received golf training (14.3%), but none played at a competitive level (0%); however, a single participant had a stroke handicap of 20 but did not play regularly (7.1%). In their own sport, most competed at a national level (78.6%), two competed internationally (14.3%), and a single participant competed at a national *and* international level (7.1%). Based on sport classification guidelines (O’Connor et al. 2022), most competed in invasion‐sports (i.e., ball sports, 28.6%), travel sports (e.g., swimming or athletics, 28.6%), or another sport type (e.g., fencing, 28.6%), whereas the remainder competed in court sports (e.g., tennis, 14.3%). The inclusion of a skill‐matched control group to experts allowed for an evaluation of how well general co‐ordination skill or ability to perform under novel task constraints may confound the comparison of high versus low levels of task expertise between groups across different task environments (i.e., VR and the real‐world). Unlike novices, who were split into low and high performing groups, athletes were analysed as a single group. This approach was based on the reasons that athletes were a more homogeneous group relating to motor skill proficiency compared to novices, alongside the rationale that athletes were used as a benchmark between expert and novice comparisons due to their high motor skill development but in a sport different to golf.

*Experts*. Golfers with a competitive stroke handicap of less than 10 (*n* = 5) participated in exchange for the chance to win one of two gift cards ($250AUD). The mean stroke handicap was 4.60 (SD = 3.85) and ranged from 0 to 8. Based on stroke handicap criteria outlined by Merry et al. (2022), experts were classified as: professional (≤ 0; 20%), elite (1–5; 20%) and high‐level to elite (6–10; 60%). Two of the experts were also athletes in a sport other than golf, with one competing nationally in a travel sport, and the other competing internationally in an invasion sport.

## 3 Materials

### 3.1 Real‐World Putting Task

Real‐world putts were taken on an artificial non‐directional (i.e., based on grass weave) flat putting green from 10 ft (3.05 m) using a regulation size ball towards a standard sized hole. Participants used a MultiLink PS‐1 right‐handed putter while wearing a Tobii Pro Glasses 2 eye‐tracker. All participants, including expert golfers, used the same putter and ball to ensure consistency across participants (e.g., control for weight difference between clubs). The Pro Glasses 2 used a single‐point calibration system and has a spatial accuracy of 0.5° with a trackable horizontal and vertical field of view of 160°. The eye‐tracker was running off a Dell Latitude 5500 laptop and was controlled through Tobii ProController software. The final resting position of the ball following each putt was photographed using a Sony HDR‐CX405HD camera mounted to an overhead projector beam. Line, length and radial error (RE) were calculated, overcoming limitations associated with using RE alone, which confounds line and length (Neumann and Thomas 2008). Gaze data were manually coded using ELAN (Wittenburg et al. 2006).

### 3.2 Virtual Reality Putting Task

The VR simulation used was developed by D. J. Harris et al. (2021), and was presented using a HTC Vive Pro Eye, a six degrees of freedom unit with a 110° diagonal and 98° horizontal and vertical field of view. The in‐built Tobii eye‐tracker had a sampling binocularity of 120 Hz (i.e., 60 per eye), a resolution of 1440 × 1600 pixels per eye (90 Hz refresh rate) and is accurate between 0.5° and 1° of visual angle. The computer used was a Dell Precision 3630 running an Intel i7 processor and UHD graphics card. Visual (ball location) and auditory (ball being hit) feedback was provided. No other sensory information (e.g., haptic) was provided. A real‐world golf club was affixed with a HTC Vive Tracker (3.0) for VR putts. The virtual ball (4.27 cm) and hole (10.80 cm) were PGA regulation size, and putts were taken from 10 ft (3.05 m). Putting performance and eye‐gaze data were extracted through simulation output using the open‐source statistical software R Studio (R Core Team 2022).

### 3.3 Self‐Report Measures

*Simulator Task Load Index (SIM‐TLX)*. The SIM‐TLX was used to measure subjective workload (D. Harris, Wilson, and Vine 2020). The nine items included: physical, mental and temporal demands, frustration, task complexity, situational stress, distractions, perceptual strain, and task control. Response anchors ranged from very low (0) to very high (21). Instead of multiplying each subscale score by a weighted value (through pairwise comparisons), unweighted values were used for analysis, as to not confound actual and perceived workload (Arthur et al. 2023). The total scale score was calculated from the composite arithmetic mean. Internal reliability was high (*α* = 0.88).

*Simulator Sickness Questionnaire (SSQ)*. The 16‐item SSQ measured three aspects of sickness symptomology; nausea, disorientation, and oculomotor (Kennedy et al. 1993) on a 4 point scale ranging from none (0) to severe (3). The SSQ has been used prior to assess CS in a virtual walking task (J. Lee et al. 2017). Mean scale and subscale scores were used for analysis and the reliability was high (*α* = 0.85).

*Reality Judgement and Presence Questionnaire (RJPQ)*. The RJPQ was used to measure environmental perception in VR over three subscales; reality judgement, internal and external correspondence, and attention and absorption (Baños et al. 2000). Each of the 18 items had response options ranging from not at all (0) to absolutely (10). The total scale and subscale mean scores were used for analyses. The RJPQ is a commonly used measure of presence in VR (Skarbez et al. 2017). Internal reliability was high on the scale total (*α* = 0.90), and each subscale had acceptable reliability; reality judgement (*α* = 0.90), presence (*α* = 0.82), and attention and absorption (*α* = 0.70).

### 3.4 Procedure

Participants entered the lab on a single occasion and read an information sheet and signed an informed consent (ethical approval granted by the host institution; 2021/367) in accordance with the Declaration of Helsinki (World Medical Association 2013). Each participant then answered questions regarding their demographic characteristics, VR use, and experience in sports, followed by the SSQ (baseline). Participants completed 30 putts in the real‐world or in VR (counterbalanced between participants). In the real‐world, participants were fitted with the Pro Glasses 2 and calibrated using Tobii ProController prior to putting. Following each putt, the experimenter would collect the ball and place it in the putting position for the next trial. Participants completed the SSQ and SIM‐TLX following real‐world putting. Self‐report data were collected electronically using Inquisit 6 (Inquisit 6 (Millisecond) 2024). When putting in VR, the participants were fitted with the Vive Pro Eye and the headset was adjusted for optimal fit and for eye‐tracking (e.g., interpupillary distance) and were then calibrated using SteamVR. Following a VR putt, the next trial would be loaded, and the ball would re‐spawn from the standardised putting position. After VR trials, participants completed the SSQ, SIM‐TLX, and RJPQ. Participants completed all 30 trials in a single block but were notified that they could take a rest‐pause at any point throughout testing. Participants were not required to assume a fixed putting stance and were able to reset their position and complete a new routine each trial. Following all putts, participants were debriefed.

### 3.5 Data Scoring and Analysis

Because of space restrictions, putts in the real‐world could not exceed a length of 4.05 m (i.e., 1 m past the hole), so a RE of 1 m (100 cm) was selected as the real‐world cut‐off. In VR, the RE cut‐off was 2 m (200 cm) due to literature which indicates that RE may exceed 100 cm (D. J. Harris et al. 2021). Putts in VR beyond 2 m were excluded, due to a likely incorrect registering of the real‐club and VR ball. Following two successive > 2 m RE VR trials, the club was inspected and adjusted to check for sensor slippage, knocking, or any other confound which may contribute to inaccurate VR putts. All putts with a RE beyond 100 cm were recoded to a maximum value of 100 cm to account for the different cut‐offs between VR and the real‐world, with length adjusted accordingly (e.g., length > 405 cm recoded to 405 cm). Following a multiverse analysis, a winsorised approach was chosen as it retains information about low accuracy putts that omission would lose (i.e., only including putts with a RE < 1 m), whereas including putts with an RE of up to 2 m in VR inflated RE estimates. Overall interpretation between models were near identical (see Supporting Information S1 for a detailed comparison). Variability in performance is typically high in novice samples. To account for this, the novice participants were split into low performing novice (LPN) and high performing novice (HPN) groups using the median score of the average RE of all novices in the real‐world. Descriptive statistics for putting outcome measures are provided within the Supporting Information S1 (winsorised model for performance data used in the present study).

Based on the guidelines of J. N. Vickers (1996), eye‐gaze fixation events were defined as a steady gaze on an AOI for at least 100 ms, within 1°–3° of visual angle, with offset calculated following a saccade away from the AOI exceeding 3°. AOIs were defined as the ball, hole, club and the area between the ball and hole which is important for green reading (Campbell and Moran 2014). The number of fixations was calculated from the first look at an AOI following the start of a trial to the completion of the QE. Fixation duration was calculated as the elapsed time of gaze offset, relative to onset, whereas an AOI was defined as the ball, hole, club and area between the hole and ball (Campbell and Moran 2014). Consistent with the definition of Causer et al. (2017), QE was defined as the final fixation on an AOI before a critical task movement (e.g., the club backswing), which can extend beyond movement execution until a saccade away from the AOI (i.e., > 3° of visual angle). Cumulative dwell time (*Dwell* hereafter) was calculated as an aggregate measure of time spent looking at AOIs throughout task execution (terminating after the saccade away following the QE fixation). String Edit Distance (SED) was calculated based on Levenshtein distance, the number of changes required for a string to emulate a referent string (Yujian and Bo 2007). Based upon the QET fixation strategy proposed by Moore et al. (2012), the ‘Ball‐Hole‐QE’ fixation strategy was selected as a referent string. For example, a single fixation strategy of ‘QE’ would have an SED of two (as Ball and Hole need to be added) and ‘Hole‐Ball‐Hole‐QE’ has an SED of one (removal of first fixation towards the hole). SED has been used to calculate the difference between gaze sequences previously (Foulsham and Underwood 2008).

The design was a 2 (Environment: Real‐World, VR) × 4 (Expertise: LPN, HPN, Athlete, Expert) × 3 (Block: Block 1, Block 2, Block 3) cross‐sectional experimental. Putting block was defined as putts 1–10 (block 1), 11–20 (block 2), and 21–30 (block 3). The purpose of splitting performance by block was to examine whether oculomotor adaptations or putting performance changed throughout experimentation (Vine and Wilson 2011b). Putting performance (i.e., putts holed, RE, line, and length) and eye‐gaze behaviours (i.e., number of fixations, average fixation duration, QE, dwell, and SED) were analysed using linear mixed models (LMM's), predicted by environment, expertise, and block (fixed factors) with participant as the intercept using lme4 in R Studio (Bates et al. 2015). LMM's were used to overcome limitations of missing trial‐level eye‐tracking and putting performance data (Speelman and McGann 2013), and to reduce Type I error while maintaining statistical power (Matuschek et al. 2017), particularly when sample sizes are unbalanced (Isik et al. 2017). Model information for putting and gaze is shown in Appendix A. Non‐significant post‐hoc tests are provided within the Supporting Information S1. Performance, gaze data, and analysis code are available on OSF (https://osf.io/rgbty/).

Environment by expertise (2 × 4) ANOVAs were calculated for SSQ and SIM‐TLX scores (Type III). RJPQ total and subscale scores were compared using a one‐way ANOVA predicted by expertise. Tukey correction was applied to post‐hoc comparisons for survey data using Jamovi (Jamovi 2022). To examine the relationship between perception and performance across environments, a moderated regression analysis was conducted on RE scores predicted by QE and expertise by environment (moderator). QE was selected as the predictor, due to the relationship between QE and superior performance when putting (Vine et al. 2011a), and the relationships between a longer QE and higher levels of task expertise (Lebeau et al. 2016). Regression diagnostics were met based upon guidelines which accounts for structural collinearity inherent to moderated regression analyses (McClelland et al. 2017). Data wrangling, assumption checking and analyses were conducted using RStudio (RStudio Team 2020) and Jamovi (Jamovi 2022).

## 4 Results

### 4.1 Putting Performance

Please refer to Table 2 for putting performance outcome ANOVA table, including holed putts, RE, line, and length of putt.

**TABLE 2:** ANOVA tables for putting performance outcomes, including hold putts, RE, line of putt, and length of putt.

Statistical model	df numerator	df denominator	*F*	*p* value
Holed putts				
Environment × expertise × block	6	263.67	0.88	0.507
Environment × expertise	3	266.04	18.33	< 0.001
Environment × block	2	263.63	6.00	0.003
Expertise × block	6	263.67	0.51	0.800
Environment	1	265.02	407.73	< 0.001
Expertise	3	53.59	9.60	< 0.001
Block	2	263.63	6.15	0.002
Radial error				
Environment × expertise × block	6	2626.44	0.18	0.983
Environment × expertise	3	2653.61	30.95	< 0.001
Environment × block	2	2623.73	2.86	0.057
Expertise × block	6	2626.49	0.92	0.479
Environment	1	2649.19	490.59	< 0.001
Expertise	3	51.23	19.76	< 0.001
Block	2	2624.11	4.57	0.010
Line of putt				
Environment × expertise × block	6	2606.80	1.13	0.341
Environment × expertise	3	2624.35	9.12	< 0.001
Environment × block	2	2605.71	0.09	0.914
Expertise × block	6	2606.64	0.71	0.639
Environment	1	2619.51	135.39	< 0.001
Expertise	3	44.66	6.27	0.001
Block	2	2605.70	1.45	0.235
Length of putt				
Environment × expertise × block	6	2618.60	2.35	0.029
Environment × expertise	3	2644.17	16.49	< 0.001
Environment × block	2	2616.48	0.26	0.771
Expertise × block	6	2618.46	1.12	0.348
Environment	1	2638.72	140.77	< 0.001
Expertise	3	49.12	5.18	0.003
Block	2	2616.63	1.81	0.165

*Holed Putts*. When putting in the real‐world, experts holed more putts than athletes (*t* (115) = 3.47, *p* = 0.016, *d* = 1.33), HPN (*t* (115) = 3.09, *p* = 0.050, *d* = 1.08), and LPN (*t* (116) = 7.21, *p* < 0.001, *d* = 2.51), while athletes (*t* (117) = 4.39, *p* = 0.001, *d* = 1.18) and HPN (*t* (117) = 6.66, *p* < 0.001, *d* = 1.44) holed more putts than LPN. All groups holed more putts in the real‐world compared to VR: experts (*t* (264) = 10.16, *p* < 0.001, *d* = 3.71), athletes (*t* (264) = 9.54, *p* < 0.001, *d* = 2.46), HPN (*t* (264) = 16.07, *p* < 0.001, *d* = 2.88), and LPN (*t* (275) = 7.40, *p* < 0.001, *d* = 1.33). There were no differences across expertise levels in the average number of holed putts in VR (all *t*'s < 0.69, *p*'s > 0.997, *d*'s < 0.24). See Table 3 for the average number of holed putts by expertise in the real‐world and in VR.

**TABLE 3:** Average number of holed putts by expertise in the real‐world and in VR.

Putting environment	LPN		HPN		Athletes		Experts	
	Mean	SD	Mean	SD	Mean	SD	Mean	SD
Real‐world	5.33	3.04	10.30	2.87	9.40	5.40	14.00	4.74
VR	0.71	1.06	0.38	0.67	0.90	0.88	1.20	0.84

Abbreviations: HPN = high performing novices, LPN = low performing novices.

**FIGURE 1:**
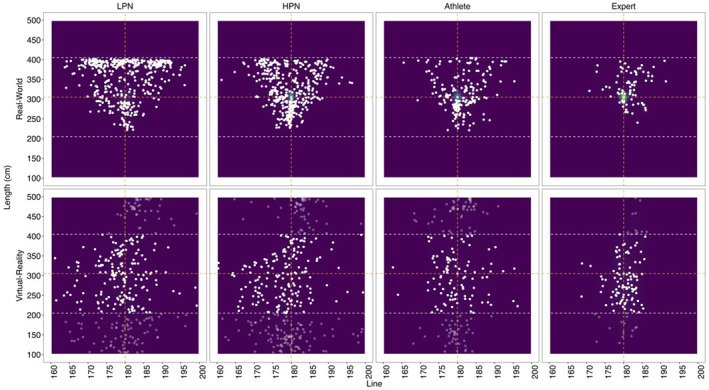
The final ball position across real‐world and VR in the low performing novices (LPN), high performing novices (HPN), athlete and expert groups, where putts which were recoded to adjust for RE and length have reduced colour opacity.

*Radial Error*. In the real‐world, experts (*t* (100) = −8.62, *p* < 0.001, *d* = −1.39), athletes (*t* (108) = −8.44, *p* < 0.001, *d* = −0.75) and HPN (*t* (107) = −9.32, *p* < 0.001, *d* = −0.66) had a lower RE than LPN. In VR, experts had a lower RE than HPN (*t* (123) = −4.43, *p* < 0.001, *d* = −0.53), but not any other group (all *t*'s < 3.02, *p*'s > 0.059, *d*'s < 0.40). All groups had a higher RE in VR when environments were compared separately for each group (all *t*'s > 6.23, *p*'s < 0.001, *d*'s > 0.42). RE was higher at block 1 than block 3 (*t* (2624) = 2.84, *p* = 0.013, *d* = 0.15). No other effects were statistically significant (all *F*'s < 2.86, *p*'s > 0.058). Refer to Figure 1 for the final ball landing positions in VR and the real‐world for LPN, HPN, athletes and experts.

*Line*. In VR, experts had a more accurate putt line compared to HPN (*t* (57) = 3.67, *p* = 0.012, *d* = 0.78) and LPN (*t* (57) = 4.79, *p* < 0.001, *d* = 1.02). Athletes (*t* (62) = 3.54, *p* = 0.17, *d* = 0.59) also showed greater line accuracy than LPN. There were no between‐groups differences in the real‐world. Except experts, all groups (all *t*'s > 4.97, *p*'s < 0.001, *d*'s > 0.45), had a superior putting line in the real‐world compared to VR. No other effects were significant (all *F*'s < 1.45, *p*'s > 0.235).

**FIGURE 2:**
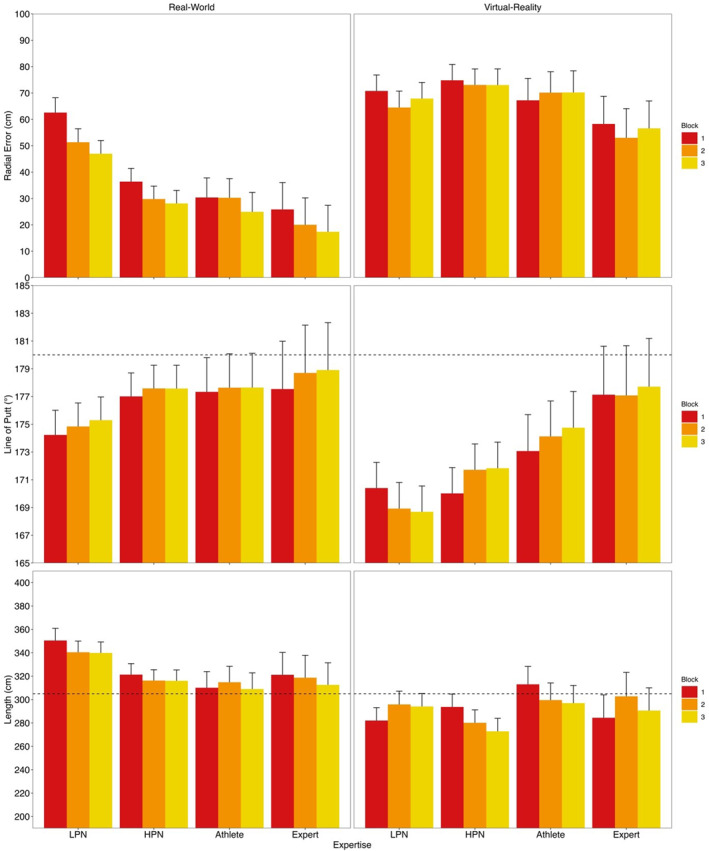
Putting outcome measures for low performing novices (LPN), high performing novices (HPN), athletes and experts in the real‐world and in VR by putting block. The dashed line indicates the hole position with the upper 95% CI shown.

*Length*. In VR, no block, expertise or interaction effects were statistically significant (all *F*'s < 2.27, *p's* > 0.095). In the real‐world, only the main effect of expertise was statistically significant. Experts (*t* (50) = −3.56, *p* = 0.004, *d* = −0.80), athletes (*t* (52) = −5.69, *p* < 0.001, *d* = −0.99) and HPN (*t* (52) = −5.63, *p* < 0.001, *d* = −0.79) had a shorter putt length than LPN. No other effects were significant (all *F*'s < 1.81, *p*'s > 0.165). See Figure 2 for putting outcome measures for LPN, HPN, athletes and experts in the real‐world and in VR.

### 4.2 Eye‐Gaze Measures

Refer to Table 4 for the ANOVA tables for eye‐gaze outcomes, including QE, number of fixations, mean fixation duration, dwell and SED.

**TABLE 4:** ANOVA tables for eye‐gaze variables, including quiet eye, number of fixations, mean fixation duration, cumulative dwell time, and string edit distance.

Statistical model	df numerator	df denominator	*F*	*p* value
Quiet eye				
Environment × expertise × block	6	2083.02	0.30	0.935
Environment × expertise	3	2120.44	16.97	< 0.001
Environment × block	2	2082.26	0.08	0.919
Expertise × block	6	2082.77	0.48	0.821
Environment	1	2121.68	226.38	< 0.001
Expertise	3	51.89	1.55	0.213
Block	2	2082.03	2.41	0.090
Number of fixations				
Environment × expertise × block	6	2184.50	1.72	0.113
Environment × expertise	3	2216.14	2.33	0.073
Environment × block	2	2183.91	0.51	0.600
Expertise × block	6	2184.37	2.57	0.018
Environment	1	2215.81	8.97	0.003
Expertise	3	52.63	0.59	0.621
Block	2	2183.83	2.85	0.058
Mean fixation duration				
Environment × expertise × block	6	2082.22	0.86	0.526
Environment × expertise	3	2118.41	10.99	< 0.001
Environment × block	2	2081.51	0.20	0.816
Expertise × block	6	2082.00	1.15	0.331
Environment	1	2118.91	211.61	< 0.001
Expertise	3	51.48	1.62	0.195
Block	2	2081.31	1.17	0.310
Cumulative dwell				
Environment × expertise × block	6	2083.73	1.49	0.178
Environment × expertise	3	2115.95	1.25	0.290
Environment × block	2	2083.11	1.32	0.267
Expertise × block	6	2083.54	2.11	0.049
Environment	1	2115.65	288.37	< 0.001
Expertise	3	53.57	2.60	0.062
Block	2	2082.95	2.14	0.118
String edit distance				
Environment × expertise × block	6	2187.36	0.75	0.613
Environment × expertise	3	2223.99	5.41	0.001
Environment × block	2	2186.21	1.98	0.138
Expertise × block	6	2186.96	1.22	0.291
Environment	1	2234.34	84.60	< 0.001
Expertise	3	52.77	1.05	0.379
Block	2	2185.96	0.25	0.783

*Quiet Eye Duration*. There was no difference between expertise groups within VR or the real‐world, but a larger QE in VR than the real‐world for all groups: expert (*t* (2080) = 3.31, *p* = 0.022, *d* = 0.42), athlete (*t* (2134) = 12.13, *p* < 0.001, *d* = 1.40), HPN (*t* (2132) = 10.27, *p* < 0.001, *d* = 0.80) and LPN (*t* (2134) = 5.89, *p* < 0.001, *d* = 0.47). No other effects were statistically significant (all *F*'s < 2.41, *p*'s > 0.090).

*Number of Fixations*. The expertise × block interaction was statistically significant (*F* (62,184.4) = 2.57, *p* = 0.018), although no post‐hoc tests which compared groups separately for each block were significant (all *t*'s < 3.24, *p*'s > 0.055, *d*'s < 0.69). There were more fixations in the real‐world than in VR (*t* (2216) = 3.00, *p* = 0.003, *d* = 0.15). No other effects were statistically significant (all *F*'s < 2.85, *p*'s > 0.058). Data for eye‐gaze measures are provided within the Supporting Information S1.

**FIGURE 3:**
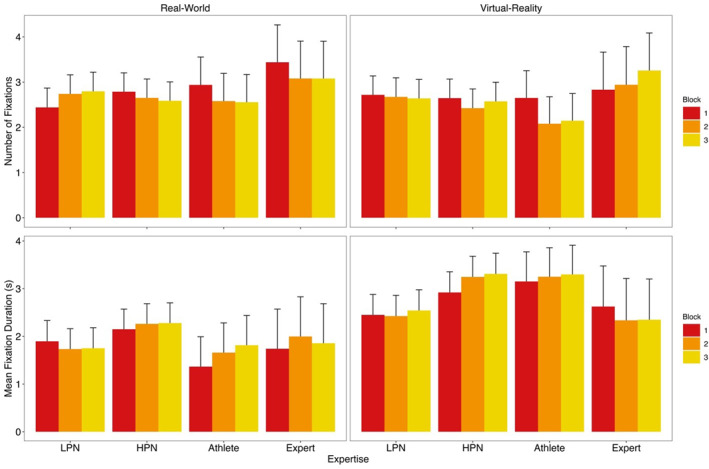
Number of fixations and average fixation duration for low performing novices (LPN), high performing novices (HPN), athletes and experts in the real‐world and VR by putting block with the upper 95% CI shown.

*Mean Fixation Duration*. Fixation duration was larger in VR compared to the real‐world for all groups while there was no difference between groups within each putting environment: expert (*t* (2080) = 3.50, *p* = 0.011, *d* = 0.45), athlete (*t* (2134) = 11.05, *p* < 0.001, *d* = 1.27), HPN (*t* (2130) = 9.37, *p* < 0.001, *d* = 0.73) and for LPN (*t* (2121) = 6.73, *p* < 0.001, *d* = 0.53). No other effects were significant (all *F*'s < 1.62, *p*'s > 0.195). See Figure 3 for number of fixations and mean fixation duration for LPN, HPN, athletes and experts in the real‐world and in VR.

**FIGURE 4:**
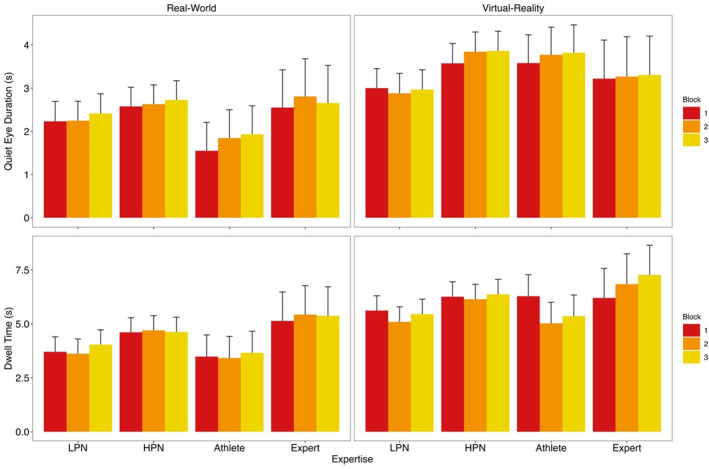
Quiet eye duration and cumulative dwell time for low performing novices (LPN), high performing novices (HPN), athletes and experts in the real‐world and in VR by putting block with the upper 95% CI shown.

*Dwell Time*. The expertise × block interaction was significant; however, no post‐hoc tests were statistically significant (all *t*'s < 2.76, *p*'s > 0.200, *d*'s < 0.35). Dwell was longer in VR compared to the real‐world (*t* (2116) = 16.98, *p* < 0.001, *d* = 0.87). No other effects were significant (all *F*'s < 2.60, *p*'s > 0.062). See Figure 4 for QE duration and dwell time for LPN, HPN, athletes, and experts in the real‐world and in VR.

*String Edit Distance*. Table 5 shows the proportion of gaze strategies used in VR and in the real‐world for LPN, HPN, athletes, and experts. There was no difference between expertise groups when compared separately for the VR and real‐world environments. In VR, compared to the real‐world, SED was longer for experts (*t* (2183.2) = 3.91, *p* = 0.002, *d* = 0.48), athletes (*t* (2208) = 6.81, *p* < 0.001, *d* = 0.75) and LPN (*t* (2235) = 4.62, *p* < 0.001, *d* = 0.35), but not for HPN. No other effects were statistically significant (all *F*'s < 1.98, *p*'s > 0.138).

**TABLE 5:** Proportion of gaze strategies used in VR and in the real‐world for low performing novices (LPN), high performing novices (HPN), athletes, and expert groups.

Sequence	LPN		HPN		Athlete		Expert	
	*n*	%	*n*	%	*n*	%	*n*	%
Real‐world								
QE	108	29.11	152	33.63	34	17.89	12	8.22
H‐QE	17	4.58	2	0.44	16	8.42	0	0
B‐H‐QE	182	49.06	231	51.11	116	61.05	107	73.29
H‐B‐H‐QE	11	2.96	5	1.11	11	5.79	1	0.68
B‐H‐B‐H‐QE	44	11.86	53	11.73	13	6.84	26	17.81
Other	9	2.43	9	1.99	0	0	0	0
Total	371	100	452	100	190	100	146	100
Virtual reality								
QE	92	25.41	140	35.53	104	47.71	26	20.47
H‐QE	69	19.06	36	9.14	22	10.09	18	14.17
B‐H‐QE	92	25.41	118	29.95	62	28.44	50	39.37
H‐B‐H‐QE	53	14.64	48	12.18	15	6.88	20	15.75
B‐H‐B‐H‐QE	24	6.63	17	4.31	9	4.13	9	7.09
Other	32	8.84	35	8.88	6	2.75	4	3.15
Total	362	100	394	100	218	100	127	100

Abbreviations: B = ball, H = hole, HPN = high performing novices, LPN = low performing novices, QE = quiet eye.

### 4.3 Perception and Action: Quiet Eye and Putting Success by Environment

**FIGURE 5:**
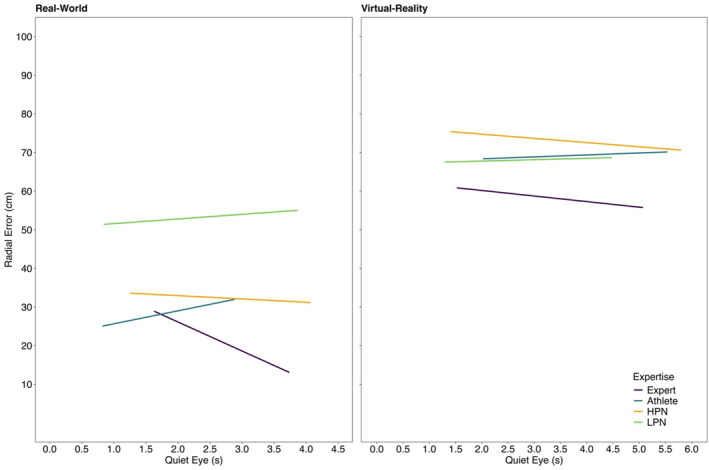
The relationship between quiet eye duration on radial error for low performing novices (LPN), high performing novices (HPN), athletes, and experts in VR and in the real‐world.

The model was statistically significant (*F* (15,2142) = 43.00, *p* < 0.001) and explained 22.61% of model variance (Adjusted *R* ^*2*^). The between‐subjects interaction of environment and expertise was statistically significant (*F* (3,2142) = 21.92, *p* < 0.001). In VR, neither QE nor QE × expertise interaction was statistically significant (all *F*'s < 0.56, *p*'s > 0.602). For real‐world putts, the expertise and environment interaction was significant (*F* (3,1151) = 4.16, *p* = 0.006). Simple slopes analysis revealed that a 1‐unit increase in QE did not predict a change in RE for the LPN (*β* = 1.20, *p* = 0.280), HPN (*β* = −0.85, *p* = 0.430), or athlete groups (*β* = 3.36, *p* = 0.140). For experts, RE was reduced by 7.51 cm for every 1‐s increase in QE (*β* = −7.51, *p* < 0.001). Please see Figure 5 for the relationship between QE on RE in the real‐world and in VR for LPN, HPN, athlete and expert groups.

### 4.4 Experience of VR: Cybersickness, Workload and Subjective Experience

#### 4.4.1 Simulator Sickness Questionnaire

The effect of expertise was significant on total SSQ (*F* (3,166) = 4.75, *p* = 0.003, *η*_p_^2^ = 0.08) and on the oculomotor subscale (*F* (3,166) = 5.04, *p* = 0.002, *η*_p_^2^ = 0.08). Experts reported less symptoms than LPN (all *t*'s > 2.65, *p*'s < 0.045, *d*'s > 0.93). There was an effect of environment, where scores were higher in VR compared to the real‐world, for the total SSQ (*F* (2,166) = 14.07, *p* < 0.001, *η*_p_^2^ = 0.14) and on each subscale: disorientation (*F* (2,166) = 9.50, *p* < 0.001, *η*_p_^2^ = 0.10), nausea (*F* (2,166) = 12.65, *p* < 0.001, *η*_p_^2^ = 0.13) and oculomotor (*F* (2,166) = 14.59, *p* < 0.001, *η*_p_^2^ = 0.15). No environment × expertise interactions were significant (all *F*'s < 0.92, *p*'s > 0.436, *η*_p_^2^'s < 0.02).

#### 4.4.2 Simulator Task Load Index

On perceptual strain, the environment × expertise interaction was statistically significant (*F* (3,114) = 2.99, *p* = 0.034, *η*_p_^2^ = 0.07). In VR, athletes reported higher strain than HPN (*t* (114) = 3.76, *p* = 0.006, *d* = 1.30), and compared to their own real‐world scores (*t* (114) = 5.03, *p* < 0.001, *d* = 1.90). On stress, there was an effect of expertise (*F* (3,114) = 2.73, *p* = 0.047, *η*_p_^2^ = 0.07), where athletes reported higher stress than HPN (*t* (114) = 2.75, *p* = 0.035, *d* = 0.67). There was an effect of environment, where higher scores were reported following VR compared to the real‐world on the total scale score, mental demands, physical demands, frustration, task complexity, stress, distractions and difficulties with task control (all *F*'s > 3.05, *p*'s < 0.031, *η*_p_^2^'s > 0.07). Refer to Table 6 for SSQ and SIM‐TLX scale and subscale scores by expertise groups within the real‐world and in VR.

**TABLE 6:** Simulator sickness questionnaire and SIM‐TLX scores across expertise levels in VR and in the real‐world.

Scale	LPN		HPN		Athlete		Expert	
	Mean	SD	Mean	SD	Mean	SD	Mean	SD
Real‐world								
Total SSQ	0.19	0.21	0.16	0.13	0.06	0.10	0.01	0.03
Nausea	0.15	0.18	0.16	0.16	0.03	0.10	0.03	0.06
Disorientation	0.11	0.16	0.08	0.11	0.03	0.06	0.00	0.00
Oculomotor	0.32	0.36	0.27	0.24	0.11	0.20	0.00	0.00
Total SIM‐TLX	3.81	2.43	3.39	2.47	3.94	2.28	2.13	1.22
Mental	3.33	2.22	4.05	3.97	4.86	3.59	1.80	1.30
Physical	3.86	3.20	3.81	3.57	3.29	3.36	1.20	0.45
Stress	3.53	3.12	3.05	3.22	4.29	2.97	3.60	4.16
Complexity	3.62	3.40	4.33	4.61	4.64	4.29	3.00	4.47
Frustration	4.95	3.75	2.90	3.19	5.21	3.72	2.20	1.64
Distractions	3.24	2.63	3.00	2.95	2.93	2.92	1.40	0.89
Strain	3.43	2.79	2.81	2.02	2.43	2.06	2.60	2.30
Temporal	4.19	4.43	3.05	3.34	4.00	2.75	2.00	2.24
Task control	4.19	4.51	3.48	4.31	3.86	3.61	1.40	0.55
Virtual reality								
Total SSQ	0.47	0.44	0.32	0.19	0.29	0.23	0.19	0.16
Nausea	0.40	0.42	0.27	0.22	0.21	0.21	0.29	0.20
Disorientation	0.42	0.51	0.22	0.21	0.22	0.23	0.09	0.13
Oculomotor	0.69	0.53	0.51	0.31	0.46	0.40	0.31	0.40
Total SIM‐TLX	8.08	3.38	6.26	3.23	9.17	3.22	7.07	4.67
Mental	9.81	5.45	6.76	4.97	10.36	5.06	9.00	8.57
Physical	6.91	5.40	5.62	5.43	5.43	4.69	7.00	8.22
Stress	8.00	5.52	4.05	4.41	9.57	5.15	6.00	6.56
Complexity	7.76	5.42	6.71	6.14	8.43	6.45	6.00	8.46
Frustration	10.10	5.92	7.95	5.77	10.50	5.59	10.80	8.50
Distractions	5.86	4.89	3.53	2.91	7.43	5.72	2.20	2.68
Strain	6.43	4.13	4.67	4.50	9.50	5.26	4.80	6.38
Temporal	4.81	4.03	4.24	4.40	5.36	3.86	3.60	4.34
Task control	13.05	5.55	11.86	6.67	16.00	4.00	14.20	7.69

Abbreviations: HPN = high performing novices, LPN = low performing novices.

#### 4.4.3 Reality Judgement and Presence Questionnaire

Using a one‐way ANOVA, there was no effect of condition on the RJPQ total scale or subscales (all *F*'s < 1.47, *p*'s > 0.232, *η*_p_^2^'s < 0.07). See Table 7 for RJPQ scale and subscale scores by expertise level.

**TABLE 7:** Reality judgement and presence questionnaire in VR by expertise level.

Outcome	LPN		HPN		Athlete		Expert	
	Mean	SD	Mean	SD	Mean	SD	Mean	SD
Total	5.28	1.40	5.42	1.70	4.87	1.46	4.96	1.26
Attention and absorption	6.35	1.56	6.36	1.79	5.80	1.97	5.45	2.23
Presence	4.98	1.87	5.86	1.64	5.62	1.54	5.09	1.35
Reality judgement	4.98	1.56	4.46	2.23	3.57	2.07	4.54	1.75

Abbreviations: HPN = high performing novices, LPN = low performing novices.

## 5 Discussion

The purpose of the present study was to compare visuomotor co‐ordination and putting performance in VR and in the real‐world for different levels of expertise while including an athlete control group (i.e., athletes in another sport). Novice participants were also divided into those who showed relatively low or high putting accuracy in the real world. In the real‐world, experts, athletes and HPN had a lower RE than LPN, who had a longer putt length than all other groups. In the real‐world, putt length was longer, putting line was more accurate, and RE was lower than in VR, which indicates that performance overall was improved in the real‐world. This result was supported by the average number of holed putts, which was higher across all levels of expertise in the real‐world compared to VR. In VR, the expert group had reduced RE than the HPN and a more accurate line than the LPN and HPN, which suggests that an expertise specific performance advantage over other sport athletes was not evident, and may not be present in comparison to some novice samples (i.e., LPN). Compared to the real‐world, people made fewer fixations in VR, but these fixations tended to be longer, and the pattern of fixations had less consistency (i.e., as measured through SED). These findings suggest that evaluating skill performance specifically in VR may have limitations compared to testing within the real‐world performance environment. This was supported by QE regression findings, as there was no relationship between QE and RE in VR, whereas QE predicted reduced RE for experts in the real‐world. This may suggest that perception and action may be poorly coupled in VR as compared to the real‐world (Düking et al. 2018; Giesel et al. 2020; D. J. Harris et al. 2019).

Hypothesis 1, which predicted superior performance for experts in VR and in the real‐world, was partially supported. In the real‐world, experts had a lower RE than LPN while there were no differences in putt line, and a shorter putt length than LPN. In VR, experts had a lower RE than HPN, improved line compared to HPN and LPN; however, there were no differences in putt length between groups. The findings suggest that experts performed similarly to athletes or in some cases a novice subgroup in the real‐world and in VR, but instead showed their expertise in maintaining consistent performance across environments (e.g., equivalent putt line in VR compared to the real‐world). This finding may indicate that at least some real‐world specific skills may transfer to VR, which is consistent to previous VR golf putting studies (D. J. Harris et al. 2021). Although some aspects of expertise may translate from the real‐world to VR, an important caveat is that all groups reduced in their average number of holed putts in VR, and there were no group differences in VR by expertise, despite experts holing more putts than all other groups in the real‐world, and athletes and holing more putts than LPN. This finding is consistent with previous research which suggests that performance in general, may be less accurate in VR as compared to the real‐world (D. J. Harris et al. 2021). The lack of holed putts, irrespective of expertise, may have negative impacts upon learning, which could indicate that VR may not be particularly well suited for practicing visuomotor skills. For example, feedback about task success is important for reinforcing movement accuracy and consistency (Sullivan et al. 2008), promote the development of cognitive strategies related to the task such as rules and principles (Wilkinson et al. 2015), and for the development of feedforward motor control processes which are important for generalisation of learning (Raichin et al. 2021). For these reasons, VR may not always be appropriate for the practice of real‐world motor skills, and nuance is required to understand for which tasks VR‐based practice may be beneficial for (D. J. Harris et al. 2021).

All putting outcome measures were improved in the real‐world compared to VR, which supports Hypothesis 2, and suggests that VR may be a more difficult environment to successfully co‐ordinate movement (Giesel et al. 2020; Harris et al., 2019). Difficulties with visuomotor coordination within VR may also explain why there was evidence of a practice effect within the real‐world but not in VR. Under usual sensorimotor conditions and with enough time and trials, an improvement in performance through practice would be anticipated. There may have been too few trials for a practice effect to emerge in VR, potentially suggesting that the demands of acclimatising to VR may be too great for there to be effective learning through practice within short duration exposures. Within a training context, this could suggest that VR practice within short bursts could be an ineffective learning strategy, and may even impair real‐world performance within the short‐term, as shown by an increase in RE on the first putt back in the real‐world following VR in another study (D. J. Harris, Buckingham, et al. 2020). It may be that more sessions, or repeated practice within sessions is required to observe practice effects in VR. This insight may suggest that the learning trajectories between VR and the real‐world may be different, where benefits to performance from VR compared to the real‐world may be delayed in the early stages of skill learning. However, it is plausible that a training protocol in addition to VR practice (such as QET), may be required for performance benefits to emerge, which would suggest that VR practice may be effective if implemented alongside training which mitigates some adverse effects on visuomotor coordination when learning a skill within VR (Bennett et al. 2025).

The lack of performance improvement from VR practice could also be explained by a learning generalisation effect, where participants had more experience putting in the real‐world, making it easier to generalise their skills to the real‐world putting green, while the VR green was comparatively more dissimilar and transfer of skills was more difficult (Krakauer et al. 2006). It is also important to caveat that movement itself could be relatively consistent between the real‐world and VR, but input or outcome in VR contains error not present in the real‐world (Wang et al. 2023). For experts, QE was associated with lower RE in the real‐world, which is consistent with literature on the performance enhancing effect of QE (Lebeau et al. 2016). However, QE was not associated with RE in VR, which highlights an inherent challenge with VR, which is understanding the perception–action loop and perception–action coupling in VR (D. J. Harris et al. 2019). This may be evidenced by longer fixation duration and cumulative dwell times in VR, alongside a larger SED (in‐part underpinned by a reduction in the number of fixations used).

Hypothesis 3 was unsupported, as there was no difference between groups in QE in the real‐world or in VR. However, all groups QE was longer in VR compared to the real‐world, where the effect size was largest for athletes. Longer QE fixation times in VR may be due to visual fatigue in VR (Cazzoli et al. 2014), or a process of trying to understand the environmental mechanics (Heilmann and Witte 2021), or be caused by reduced gaze precision and impairments to information extraction (Pastel et al. 2020). There is also research which suggests that people may attend to meaningful areas in the environment for longer in VR than in the real‐world (Haskins et al. 2020), which could explain the longer QE fixation times and increased use of the QE fixation strategy in VR. It is noteworthy that eye‐movements measure overt attention, and in sports tasks, covert attention is also required to identify and track AOI's (Memmert 2009). It may be that people ‘looked’ for longer at an AOI because they were resting their gaze, but tracking information elsewhere, particularly if making a fixation to that location in VR could contribute to oculomotor discomfort, or cybersickness symptomology (Souchet et al. 2022). There were no group differences in QE duration across expertise in either putting environment, although QE duration predicted performance in the real world within the expert group. The lack of group differences may be due to group means in both the real‐world and in VR being comparable to an optimal QE duration of 2.5 s (J. N. Vickers 1992). There was also moderate variability in QE duration within each expertise group across the real‐world and VR, which may contribute to a lack of statistical difference between expertise groups in each putting setting, a finding that could reflect individual differences in optimal QE durations (Ziv and Lidor 2019).

The SSQ total and subscale scores and the SIM‐TLX total and most subscale scores were higher following VR than following real‐world putting, alongside perceptions of reduced task control. From a motivational perspective, particularly for LPN, HPN, and athletes that may not have a sport‐specific interest in golf, the increase in mental demands may lead to a reduction in effort and task engagement (Hopstaken et al. 2016). However, athletes may have entered a challenging state, which could have led to increased frustration also (Meijen et al. 2020). The cybersickness results related to higher scores in VR as compared to the real‐world for people of all expertise levels may support the theory that the accommodation–vergence conflict, where the eyes rotate based on projected image (accommodation), and not perceived location (vergence) is partly responsible for oculomotor and cybersickness symptoms in VR (Cazzoli et al. 2014; Davis et al. 2014). However, the result may also indicate that people with task‐specific oculomotor expertise (e.g., are used to performing a QE), may be somewhat inoculated to the muscular and oculomotor fatigue of VR (i.e., increased extraocular strength), which may reduce near point convergence and accommodation (Watten et al. 1994).

The putting results of athletes suggest improved performance to LPN in the real‐world, but no differences to any other group in the real‐world. The athletes were similar to novices when performing in VR. There should be advantages to performance for athletes compared to novices, including more quickly (1) learning new perception–action couplings (2) modifying pre‐existing skills to operate in a new context (Wood et al. 2020), and (3) the use of cognitive skills developed throughout their sporting careers to overcome any potential perception or action deficits inherent to VR, such as kinaesthetic imagery to help plan and/or execute movement (Richlan et al. 2023), a cognitive skill which is also shown to improve golf‐putting performance (McNeill et al. 2020). One potential explanation is that athletes could have struggled to perform these mental tasks, as VR may be processed as a distinct performance context as compared to the real‐world (Yarossi et al. 2021). It may be that the reason why experts and athletes did not decline in performance from the real‐world to VR identically, is because experts have a knowledge advantage. In combination, this may suggest that like motor skill performance, some cognitive skills may be easier to generalise from the real‐world to VR, which has implications for what type of practice is appropriate in VR, who should complete that practice, and how VR may be effectively leveraged for real‐world skill learning. For example, experts may have calculated the distance to the hole with a high level of accuracy based on previous experience (D. J. Harris et al. 2021), and with automated movement control processes, were able to maintain their relative superior performance (Wulf et al. 2001, 2010). Athletes may have had benefits to movement compared to novices, but without the specific expert knowledge to guide their top‐down movement planning and co‐ordination, their movement coordination alone was not effectively reflected within their VR performance. In future, assessing which cognitive and movement control skills are used by experts and athletes when transitioning from the real‐world to VR may help to uncover automatic behavioural adaptations and if there are any specific elements of VR which may contribute to this effect.

### 5.1 Implications, Limitations, and Areas for Future Research

Because of the limited sample size of experts, generalisation to other VR visuomotor tasks should be made with caution. Furthermore, the lack of expertise‐related differences in VR, particularly for the athlete group compared to the novice groups, could be a result of the task itself and should be considered when generalising the findings. Given the differences in QE duration times in VR compared to the real‐world, which were largest for the athlete group, future research may benefit from including a physiological measure of oculomotor symptoms, such as blink rate or saccadic amplitude over testing, which is indicative of ocular fatigue (Souchet et al. 2022). The purpose would be to assess whether a change in QE duration times when moving from the real‐world to VR (or vice versa) is due to the task itself, or residual oculomotor symptoms throughout testing which may be elongating QE duration in VR.

It is also important to consider the specific ecological context of which putts were taken within the VR and the real‐world environment. Although putts were performed within the real‐world, they were not necessarily performed within a naturalistic setting on an outdoor green where other factors that may affect performance could be present (e.g., variability in stimp, wind, and undulations in grass). Although the absence of these factors also occurred in VR, this point speaks to a wider consideration about ethology when examining behaviour between VR contexts and the real‐world, namely the trade‐off between ecological validity and experimental control (Kingstone et al. 2008). Further clarification is needed to assess the impact on external environmental factors on the generalization of VR for practice and training.

The study's findings highlight clear differences in perception–action coupling and in performance across real‐world and VR contexts. These results may suggest that using VR as a tool for people to practice and perform skills, without providing any further support or training, may not be appropriate for skills that require a high degree of visuomotor coordination. If VR is used for visuomotor skill development, including for sport performance, a recommendation may be to focus VR training on skills that are otherwise difficult to isolate and develop in the real‐world, using techniques that cannot be effectively implemented within the real‐world. This application differs from using VR as an alternative to practice within the real‐world. The use of VR may instead include augmentation of the learning environment, such as manipulating object characteristics within the virtual environment (Godse et al. 2019), *slowing down* the environment and providing performance‐related information in real‐time (Itoh et al. 2016; Wu et al. 2021), or using 3D visualisation techniques to model the optimal behaviour from the perspective of an expert (Ikeda et al. 2019). VR can also provide advantages over the real‐world in the form of skill assessment and evaluation of decision‐making processes in real‐time (Neumann et al. 2018). Future research should also evaluate the potential of hybrid reality training (i.e., VR and real‐world combined), to see how VR can be best applied for real‐world skill learning. An example of this could be for people to putt in the real‐world but receive virtual augmented visual feedback related to performance (e.g., ball speed, landing position, trajectory of putt, and accuracy), practice on different course conditions or practice across varied competition scenarios in the same physical space.

If one of the primary purposes of VR‐based training is to develop visuomotor skills (Bird 2020), the unique perceptual demands of VR may also be a direct way to train dorsal goal‐directed attention, which may benefit performance in competition environments or under pressure. There is research to indicate that VR environments can increase anxiety and lower confidence (Stinson and Bowman 2014), which was reflected in the data among the athlete sample compared to HPN with the measure of stress, which is a similar affective state to performance under pressure. Perhaps practicing in VR over time and then manipulating task demands in VR, may be one‐way to train elite performance, such as when under pressure, or with novel task needs. In any case, VR may be leveraged to help train the high‐level executive control necessary for elite performance (T. G. Lee and Grafton 2015).

### 5.2 Conclusion

The purpose of the present study was to examine how visuomotor co‐ordination may be coupled in VR and whether task expertise may affect performance outcomes. The results suggest that VR may disrupt perception–action processes for novices and athletes, but experts may use visual system control processes and prior experience to mitigate some potential adverse perceptual effects of VR, which is how their superior performance relative to novices was maintained in VR. However, although some expertise difference can be preserved between groups in VR, expert performance itself may unequivocally differ, indicating that there is still more to uncover about the unique perceptual, motor or technological demands of VR and how it affects movement planning, co‐ordination, and performance.

## Conflicts of Interest

The authors declare no conflicts of interest

## Supporting information


Supporting Information S1

## Data Availability

Data is available from the Open Science Framework (https://osf.io/rgbty/).

## Appendix A

Model fit information across linear mixed models, including for putting performance and for eye gaze behaviour.

**Table:**

Measure	Intercept	SE	95% CI		R squared (%)	
			Lower	Upper	Conditional	Marginal
Holed putts	3.10	0.39	2.36	3.84	60.71	66.09
Radial error	30.40	3.78	23.07	37.70	23.20	24.41
Line of putt	177.33	1.25	174.94	179.73	12.16	23.03
Length of putt	310.07	7.04	296.48	323.66	11.56	14.81
Quiet eye	1.55	0.33	0.91	2.19	12.67	37.04
Number of fixations	2.94	0.31	2.34	3.53	2.82	35.82
Mean fixation duration	1.37	0.31	0.76	1.97	11.97	38.55
String edit distance	0.58	0.18	0.22	0.93	5.59	20.82
Dwell looking in AOIs	3.49	0.50	2.52	4.46	15.12	43.81
