# Supplementary material for: Get in the Virtual Hole! Examination of Gaze and Performance of Experts, Athletes, and Novices While Putting in Virtual Reality and in the Real‐World
Source: Eur J Sport Sci. 2025 Aug 30;25(9):e70049. doi: 10.1002/ejsc.70049 (PMC12397985; doi:10.1002/ejsc.70049)
Supplement: Supplementary file 1 — Supporting Information S1 [file EJSC-25-e70049-s001.docx]

**Supplementary Results**

*Get in the virtual hole! Examination of gaze and performance of experts, athletes, and novices while putting in virtual reality and in the real-world*

Jayke Bennett, David Neumann, and Matthew Stainer

**Multiverse Analysis**

**Model 1:** All VR putts included with a RE < 200 cm (2 m)

**Model 2 (Subset):** All VR putts included with a RE < 100 cm (1 m)

**Model 3 (Winsorized):** All VR putts with a RE > 1 m recoded to 1 m with length adjusted accordingly

**Table S1**

Summary of similarities and differences in results across Models 1 to 3 as part of the multiverse analysis on radial error, putt line, and putt length

|  | Model 1 | Model 2 | Model 3 |
| --- | --- | --- | --- |
| Radial Error | | | |
| Env*Exp*Block |  |  |  |
| Exp*Block |  |  |  |
| Env*Block |  |  |  |
| Env*Exp | x | x | x |
| Env | x | x | x |
| Exp | x | x | x |
| Block |  | x |  |
| Line | | | |
| Env*Exp*Block |  |  |  |
| Exp*Block |  |  |  |
| Env*Block |  |  |  |
| Env*Exp | x | x | x |
| Env | x | x | x |
| Exp | x | x | x |
| Block |  |  |  |
| Length | | | |
| Env*Exp*Block |  |  |  |
| Exp*Block |  |  |  |
| Env*Block |  |  |  |
| Env*Exp | x | x | x |
| Env | x | x | x |
| Exp |  |  |  |
| Block |  |  |  |

N.B., x indicates statistical significance

**Putting Performance**

**Table S2**

Putting outcome measures across expertise by putting block in the real-world and in VR

| Measure | LPN | | HPN | | Athlete | | Expert | |
| --- | --- | --- | --- | --- | --- | --- | --- | --- |
|  | Mean | SD | Mean | SD | Mean | SD | Mean | SD |
| Real-World | | | | | | | | |
| Line |  |  |  |  |  |  |  |  |
| *Block 1* | 175.15 | 4.33 | 177.04 | 3.31 | 177.42 | 3.53 | 177.52 | 2.99 |
| *Block 2* | 175.50 | 4.36 | 177.59 | 3.18 | 177.67 | 2.74 | 178.70 | 2.14 |
| *Block 3* | 175.92 | 4.16 | 177.59 | 3.20 | 177.73 | 3.18 | 178.91 | 2.06 |
| Length |  |  |  |  |  |  |  |  |
| *Block 1* | 349.64 | 51.15 | 321.16 | 43.52 | 309.98 | 40.95 | 321.01 | 30.69 |
| *Block 2* | 340.37 | 48.61 | 316.33 | 40.29 | 314.89 | 39.54 | 318.37 | 30.27 |
| *Block 3* | 339.96 | 44.30 | 316.06 | 37.42 | 309.07 | 34.99 | 312.57 | 28.74 |
| RE |  |  |  |  |  |  |  |  |
| *Block 1* | 62.30 | 33.27 | 36.33 | 32.41 | 30.31 | 31.73 | 25.73 | 26.72 |
| *Block 2* | 51.21 | 37.00 | 29.79 | 32.17 | 30.33 | 30.09 | 19.97 | 27.99 |
| *Block 3* | 46.89 | 36.35 | 28.13 | 30.34 | 24.97 | 28.23 | 17.39 | 25.45 |
| Virtual-Reality | | | | | | | | |
| Line |  |  |  |  |  |  |  |  |
| *Block 1* | 170.93 | 15.80 | 169.96 | 13.84 | 173.31 | 9.78 | 177.14 | 2.27 |
| *Block 2* | 169.09 | 13.57 | 171.88 | 13.30 | 174.26 | 7.89 | 177.11 | 3.81 |
| *Block 3* | 169.08 | 15.27 | 171.85 | 9.85 | 175.06 | 7.47 | 177.73 | 1.80 |
| Length |  |  |  |  |  |  |  |  |
| *Block 1* | 282.91 | 75.68 | 294.23 | 81.20 | 317.43 | 110.94 | 284.53 | 81.53 |
| *Block 2* | 296.83 | 74.88 | 280.74 | 76.41 | 301.38 | 116.54 | 305.12 | 79.69 |
| *Block 3* | 293.47 | 76.46 | 273.51 | 75.45 | 299.66 | 115.39 | 290.84 | 84.78 |
| RE |  |  |  |  |  |  |  |  |
| *Block 1* | 282.91 | 75.68 | 294.23 | 81.20 | 91.00 | 63.75 | 66.84 | 50.07 |
| *Block 2* | 296.83 | 74.88 | 280.74 | 76.41 | 97.38 | 63.17 | 61.53 | 49.68 |
| *Block 3* | 293.47 | 76.46 | 273.51 | 75.45 | 96.54 | 62.38 | 66.91 | 53.08 |

N.B., RE = Radial Error; LPN = Low Performing Novices; HPN = High Performing Novices

**Eye-Gaze Behaviours**

**Table S3**

Eye-gaze measures across expertise by putting block in the real-world and in VR

| Measure | LPN | | HPN | | Athlete | | Expert | |
| --- | --- | --- | --- | --- | --- | --- | --- | --- |
|  | Mean | SD | Mean | SD | Mean | SD | Mean | SD |
| Real-World | | | | | | | | |
| N Fix |  |  |  |  |  |  |  |  |
| *Block 1* | 2.53 | 1.28 | 2.77 | 1.49 | 3.02 | 0.97 | 3.44 | 1.17 |
| *Block 2* | 2.85 | 1.57 | 2.59 | 1.37 | 2.65 | 1.08 | 3.08 | 1.09 |
| *Block 3* | 2.82 | 1.44 | 2.54 | 1.37 | 2.64 | 1.07 | 3.08 | 0.70 |
| M Fix Dur |  |  |  |  |  |  |  |  |
| *Block 1* | 1.89 | 1.48 | 2.11 | 1.33 | 1.33 | 0.82 | 1.74 | 0.98 |
| *Block 2* | 1.79 | 1.33 | 2.29 | 1.50 | 1.63 | 1.05 | 2.01 | 0.91 |
| *Block 3* | 1.85 | 1.11 | 2.29 | 1.35 | 1.78 | 1.13 | 1.86 | 0.69 |
| QE |  |  |  |  |  |  |  |  |
| *Block 1* | 2.26 | 1.37 | 2.55 | 1.34 | 1.62 | 0.95 | 2.56 | 1.01 |
| *Block 2* | 2.32 | 1.60 | 2.69 | 1.55 | 1.92 | 1.02 | 2.84 | 1.10 |
| *Block 3* | 2.49 | 1.54 | 2.75 | 1.31 | 2.01 | 1.08 | 2.66 | 1.04 |
| Dwell |  |  |  |  |  |  |  |  |
| *Block 1* | 3.88 | 2.51 | 4.55 | 2.03 | 3.71 | 1.62 | 5.13 | 0.90 |
| *Block 2* | 3.95 | 2.16 | 4.68 | 2.13 | 3.62 | 1.64 | 5.45 | 1.26 |
| *Block 3* | 4.35 | 2.29 | 4.59 | 2.26 | 3.89 | 1.60 | 5.38 | 0.99 |
| SED |  |  |  |  |  |  |  |  |
| *Block 1* | 0.92 | 0.99 | 0.97 | 1.14 | 0.53 | 0.80 | 0.77 | 0.97 |
| *Block 2* | 1.13 | 1.10 | 0.97 | 1.04 | 0.69 | 0.87 | 0.58 | 0.92 |
| *Block 3* | 0.93 | 1.12 | 1.03 | 1.02 | 0.67 | 0.90 | 0.24 | 0.66 |
| Virtual-Reality | | | | | | | | |
| N Fix |  |  |  |  |  |  |  |  |
| *Block 1* | 2.88 | 1.54 | 2.70 | 1.48 | 2.65 | 2.11 | 2.84 | 1.29 |
| *Block 2* | 2.80 | 1.55 | 2.52 | 1.48 | 2.07 | 1.32 | 2.97 | 1.17 |
| *Block 3* | 2.76 | 1.63 | 2.61 | 1.54 | 2.17 | 1.39 | 3.26 | 1.22 |
| M Fix Dur |  |  |  |  |  |  |  |  |
| *Block 1* | 2.28 | 1.33 | 2.79 | 1.64 | 3.25 | 1.71 | 2.63 | 1.82 |
| *Block 2* | 2.38 | 1.48 | 3.17 | 2.23 | 3.36 | 1.79 | 2.35 | 0.84 |
| *Block 3* | 2.48 | 1.46 | 3.15 | 2.16 | 3.35 | 1.82 | 2.38 | 1.12 |
| QE |  |  |  |  |  |  |  |  |
| *Block 1* | 2.87 | 1.61 | 3.40 | 1.87 | 3.62 | 1.64 | 3.22 | 2.05 |
| *Block 2* | 2.87 | 1.63 | 3.75 | 2.43 | 3.86 | 1.85 | 3.32 | 1.69 |
| *Block 3* | 2.93 | 1.52 | 3.66 | 2.24 | 3.84 | 1.77 | 3.36 | 1.55 |
| Dwell |  |  |  |  |  |  |  |  |
| *Block 1* | 5.55 | 2.77 | 6.08 | 2.64 | 6.33 | 3.82 | 6.20 | 2.20 |
| *Block 2* | 5.22 | 2.12 | 6.20 | 2.75 | 5.17 | 1.72 | 6.88 | 1.89 |
| *Block 3* | 5.60 | 3.77 | 6.15 | 2.48 | 5.49 | 2.05 | 7.27 | 1.84 |
| SED |  |  |  |  |  |  |  |  |
| *Block 1* | 1.18 | 1.06 | 1.16 | 1.04 | 1.32 | 1.86 | 1.02 | 0.95 |
| *Block 2* | 1.27 | 0.94 | 1.21 | 1.04 | 1.44 | 0.94 | 0.89 | 0.94 |
| *Block 3* | 1.30 | 1.06 | 1.35 | 0.92 | 1.38 | 0.86 | 1.07 | 0.88 |

N.B., N Fix = Number of Fixations; M Fix Dur = Mean Fixation Duration; QE = Quiet Eye; Dwell = Dwell in AOIs; SED = String Edit Distance; LPN = Low Performing Novices; HPN = High Performing Novices

**Figure S1**

String edit distance across expertise by putting block in VR and in the real-world


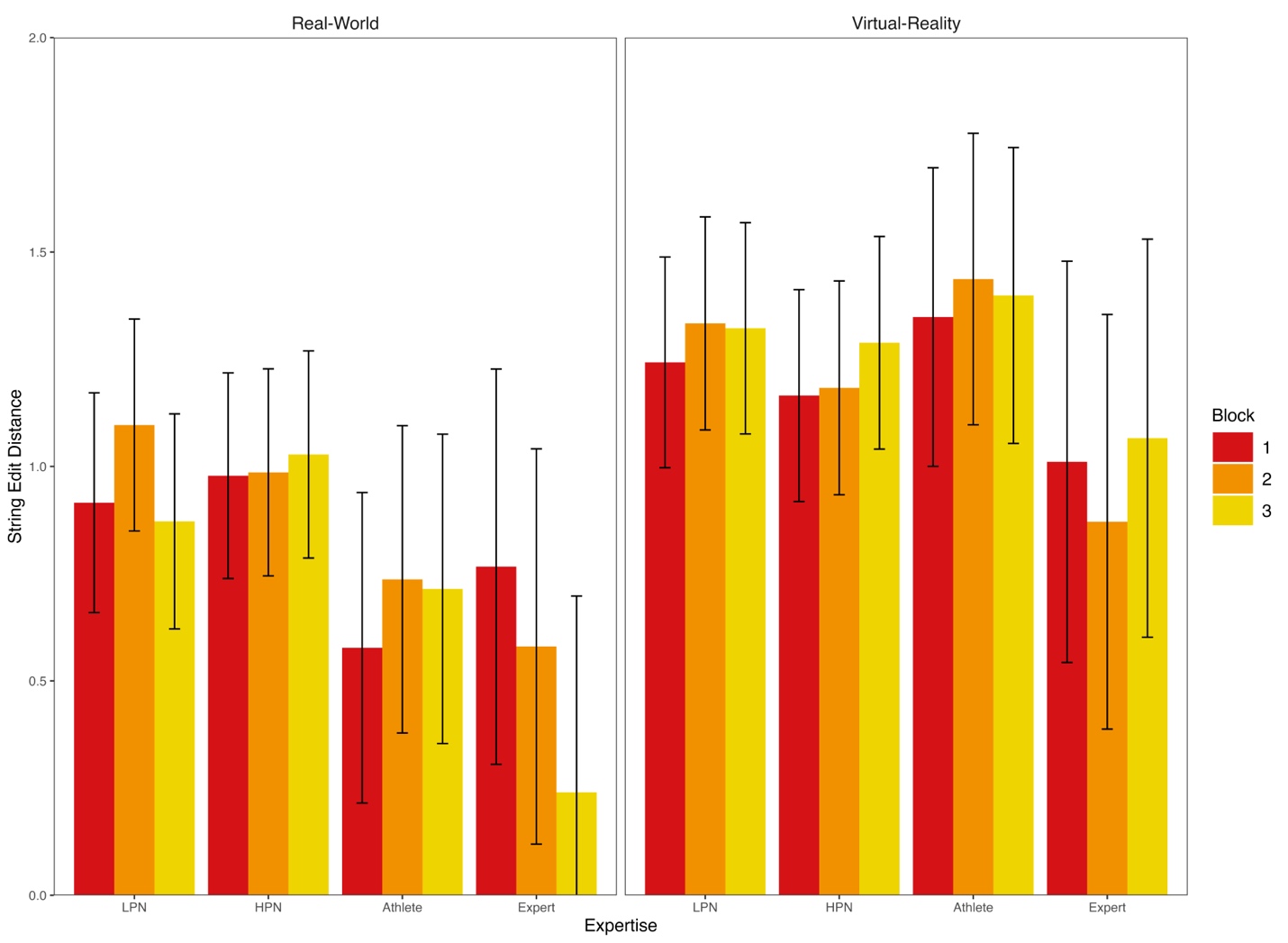


**Post-Hoc Comparisons**

**Radial Error**

**Table S4**

Tukey comparisons on RE for between each level of expertise on VR putts

| Expertise | | *df* | *t* | *p value* | *d* |
| --- | --- | --- | --- | --- | --- |
| Virtual Reality | | | | | |
| Expert | Athlete | 121 | -3.02 | 0.059 | -0.39 |
| Expert | HPN | 123 | -4.43 | < .001 | -0.53 |
| Expert | LPN | 126 | -2.94 | 0.073 | -0.35 |
| Athlete | HPN | 151 | -1.36 | 0.876 | -0.13 |
| Athlete | LPN | 155 | 0.45 | 0.999 | 0.04 |
| HPN | LPN | 184 | 2.15 | 0.390 | 0.18 |

N.B., LPN = Low Performing Novices; HPN = High Performing Novices

**Number of Fixations**

**Table S5**

Tukey comparisons on number of fixations for each expertise group across putting blocks

| Blocks | | *df* | *t* | *p value* | *d* |
| --- | --- | --- | --- | --- | --- |
| Expert | | | | | |
| Block 1 | Block 2 | 2182 | 0.70 | 0.999 | 0.11 |
| Block 1 | Block 3 | 2182 | -0.19 | 0.999 | -0.03 |
| Block 2 | Block 3 | 2182 | -0.90 | 0.999 | -0.13 |
| Athlete | | | | | |
| Block 1 | Block 2 | 2183 | 3.24 | 0.055 | 0.39 |
| Block 1 | Block 3 | 2183 | 3.06 | 0.094 | 0.38 |
| Block 2 | Block 3 | 2183 | -0.15 | 0.999 | -0.02 |
| HPN | | | | | |
| Block 1 | Block 2 | 2185 | 1.77 | 0.833 | 0.15 |
| Block 1 | Block 3 | 2185 | 1.35 | 0.972 | 0.12 |
| Block 2 | Block 3 | 2185 | -0.43 | 0.999 | -0.04 |
| LPN | | | | | |
| Block 1 | Block 2 | 2187 | -1.20 | 0.989 | -0.11 |
| Block 1 | Block 3 | 2187 | -1.30 | 0.979 | -0.12 |
| Block 2 | Block 3 | 2187 | -0.11 | 0.999 | -0.01 |

N.B., LPN = Low Performing Novices; HPN = High Performing Novices

**Table S6**

Tukey comparisons on number of fixations between expertise groups at each putting block

| Expertise | | *df* | *t* | *p value* | *d* |
| --- | --- | --- | --- | --- | --- |
| Block 1 | | | | | |
| Expert | Athlete | 59.9 | 0.70 | 0.999 | 0.29 |
| Expert | HPN | 59.1 | 0.94 | 0.998 | 0.36 |
| Expert | LPN | 59.9 | 1.25 | 0.982 | 0.47 |
| Athlete | HPN | 63.3 | 0.22 | 0.999 | 0.07 |
| Athlete | LPN | 64.8 | 0.62 | 0.999 | 0.18 |
| HPN | LPN | 65.3 | 0.49 | 0.999 | 0.12 |
| Block 2 | | | | | |
| Expert | Athlete | 60.2 | 1.39 | 0.962 | 0.58 |
| Expert | HPN | 60.1 | 1.06 | 0.995 | 0.40 |
| Expert | LPN | 60.6 | 0.68 | 0.999 | 0.26 |
| Athlete | HPN | 62.6 | -0.60 | 0.999 | -0.18 |
| Athlete | LPN | 63.5 | -1.09 | 0.994 | -0.32 |
| HPN | LPN | 64.9 | -0.61 | 0.999 | -0.14 |
| Block 3 | | | | | |
| Expert | Athlete | 59.4 | 1.67 | 0.873 | 0.69 |
| Expert | HPN | 58.7 | 1.33 | 0.972 | 0.50 |
| Expert | LPN | 59.4 | 1.02 | 0.997 | 0.38 |
| Athlete | HPN | 62.9 | -0.66 | 0.999 | -0.20 |
| Athlete | LPN | 64.1 | -1.06 | 0.996 | -0.31 |
| HPN | LPN | 65.2 | -0.49 | 0.999 | -0.12 |

N.B., LPN = Low Performing Novices; HPN = High Performing Novices

**Dwell Time in AOIs**

**Table S7**

Tukey comparisons on average dwell for each expertise group across putting blocks

| Blocks | | *df* | *t* | *p value* | *d* |
| --- | --- | --- | --- | --- | --- |
| Expert | | | | | |
| Block 1 | Block 2 | 2082 | -1.52 | 0.935 | -0.24 |
| Block 1 | Block 3 | 2082 | -2.22 | 0.535 | -0.34 |
| Block 2 | Block 3 | 2082 | -0.62 | 0.999 | -0.01 |
| Athlete | | | | | |
| Block 1 | Block 2 | 2083 | 2.76 | 0.200 | 0.34 |
| Block 1 | Block 3 | 2083 | 1.55 | 0.926 | 0.20 |
| Block 2 | Block 3 | 2083 | -1.22 | 0.988 | -0.15 |
| HPN | | | | | |
| Block 1 | Block 2 | 2086 | 0.09 | 0.999 | 0.01 |
| Block 1 | Block 3 | 2086 | -0.38 | 0.999 | -0.03 |
| Block 2 | Block 3 | 2086 | -0.46 | 0.999 | -0.04 |
| LPN | | | | | |
| Block 1 | Block 2 | 2088 | 1.73 | 0.854 | 0.16 |
| Block 1 | Block 3 | 2088 | -0.47 | 0.999 | -0.04 |
| Block 2 | Block 3 | 2088 | -2.21 | 0.544 | -0.20 |

N.B., LPN = Low Performing Novices; HPN = High Performing Novices

**Table S8**

Tukey comparisons on dwell times between expertise groups at each putting block

| Expertise | | *df* | *t* | *p value* | *d* |
| --- | --- | --- | --- | --- | --- |
| Block 1 | | | | | |
| Expert | Athlete | 61.6 | 0.98 | 0.998 | 0.41 |
| Expert | HPN | 60.7 | 0.33 | 0.999 | 0.12 |
| Expert | LPN | 61.5 | 1.39 | 0.961 | 0.53 |
| Athlete | HPN | 65.2 | -0.97 | 0.998 | -0.29 |
| Athlete | LPN | 66.6 | 0.39 | 0.999 | 0.12 |
| HPN | LPN | 66.9 | 1.69 | 0.866 | 0.40 |
| Block 2 | | | | | |
| Expert | Athlete | 62.40 | 2.38 | 0.432 | 1 |
| Expert | HPN | 62.30 | 0.98 | 0.997 | 0.37 |
| Expert | LPN | 62.90 | 2.45 | 0.393 | 0.93 |
| Athlete | HPN | 64.00 | -2.12 | 0.615 | -0.62 |
| Athlete | LPN | 65.10 | -0.23 | 0.999 | -0.07 |
| HPN | LPN | 66.50 | 2.34 | 0.462 | 0.56 |
| Block 3 | | | | | |
| Expert | Athlete | 61.2 | 2.27 | 0.506 | 0.95 |
| Expert | HPN | 60.7 | 1.14 | 0.991 | 0.43 |
| Expert | LPN | 61.6 | 2.18 | 0.570 | 0.82 |
| Athlete | HPN | 64.3 | -1.75 | 0.841 | -0.51 |
| Athlete | LPN | 65.8 | -0.42 | 0.999 | -0.12 |
| HPN | LPN | 67.1 | 1.65 | 0.886 | 0.39 |

N.B., LPN = Low Performing Novices; HPN = High Performing Novices

**Reality Judgement and Presence**

**Table S9**

Reality judgement and presence total and subscale omnibus tests across expertise

| Statistical model | *df numerator* | *df denominator* | *F* | *p value* | eta squared |
| --- | --- | --- | --- | --- | --- |
| Total | 3 | 157 | 0.45 | .721 | 0.02 |
| Attention/absorption | 3 | 157 | 0.61 | .614 | 0.03 |
| Presence | 3 | 157 | 1.08 | .365 | 0.05 |
| Reality judgement | 3 | 157 | 1.47 | .232 | 0.07 |
